# Health-Related Effects of Individual and Paired Functional High-Intensity Interval Training on Body Composition, Strength and VO_2_max in Primary School Children

**DOI:** 10.3390/healthcare14101391

**Published:** 2026-05-19

**Authors:** Diego Alonso-Fernández, Rosana Fernández-Rodríguez, Pedro Docampo-Blanco, Yaiza Taboada-Iglesias

**Affiliations:** 1 Special Didactics Department, Faculty of Science Education and Sport, University of Vigo, 36005 Vigo, Spain; diego_alonso@uvigo.gal (D.A.-F.); rosanafernandez@uvigo.gal (R.F.-R.); pedro.docampo@uvigo.gal (P.D.-B.); 2 GIES-10 (Research Group on Education, Physical Activity and Health), Galicia Sur Health Research Institute (IIS), University of Vigo, 36005 Vigo, Spain

**Keywords:** HIIT, physical education, cardiorespiratory fitness, muscular strength, primary school children

## Abstract

**Highlights:**

**What are the main findings?**

- Individual functional HIIT was associated with within-group improvements in VO_2_max and strength, whereas the paired format showed smaller or non-significant adaptations.
- The paired format was associated with a progressive decline in perceived exertion across sessions.

**What are the implications of the main findings?**

- The findings suggest that maintaining sufficient perceived intensity may be important for the effectiveness of brief HIIT interventions in school settings.
- Pedagogical organization appears to influence engagement and perceived exercise intensity during HIIT activities in Physical Education.

**Abstract:**

**Background/Objectives:** In the school setting, high-intensity interval training (HIIT) has emerged as a time-efficient strategy to improve children’s physical fitness; however, different implementation modalities have not been compared. The aim of this study was to compare the effects of an individual versus paired HIIT protocol based on functional bodyweight exercises on physical fitness-related and anthropometric outcomes in primary school children. **Methods:** Sixty-one children (11.6 ± 0.3 years) participated in a 10-week experimental study with three parallel groups: individual HIIT (EG1, *n* = 21), paired HIIT (EG2, *n* = 20), and a control group (CG, *n* = 20). Although both HIIT groups performed the same bodyweight functional exercises, in EG2 the exercises required coordinated movement between the partners. The HIIT protocol was integrated into the warm-up of Physical Education (PE) classes twice per week (Tabata-type protocol; 8 × 20 s/10 s/≤8 min per session). Body composition, muscular strength, and cardiorespiratory fitness (estimated VO_2_max) were assessed at pre- and post-test, along with a rating of perceived exertion (1–10 scale) and enjoyment/motivation (1–5 scale) across several sessions (1, 7 and 14). Data were analyzed using pre-post comparisons, ANOVA, and ANCOVA models adjusted for baseline values. **Results:** Body fat percentage decreased in all groups. The individual HIIT group showed within-group improvements in VO_2_max (+5.3%, *p* < 0.001), handgrip strength (+10.1%, *p* = 0.003), and standing long jump (+4.1%, *p* = 0.033), with moderate-to-large effect sizes, whereas the paired HIIT group showed smaller and statistically non-significant changes. Between-group comparisons suggested a tendency toward greater improvements in VO_2_max and handgrip strength in the individual HIIT group compared with the paired group, although the overall ANOVA for VO_2_max was not statistically significant. Perceived exertion declined over time in the paired group but remained relatively stable in the individual group. **Conclusions:** A low-volume HIIT program performed individually was associated with improvements in several physical fitness outcomes in schoolchildren. In contrast, paired execution showed smaller and mostly non-significant changes, together with a progressive reduction in perceived intensity.

## 1. Introduction

Our children and adolescents are increasingly engaging in sedentary behaviors that lead to hypokinetic diseases such as obesity, diabetes, and metabolic syndrome, which were previously considered to be exclusive to adults [1]. In this regard, 50% of school-aged individuals spend more than two hours per day on sedentary leisure activities, largely associated with screen use [2].

Primary education represents a critical window for establishing habits that promote healthy physical fitness and body composition in children. In this context, schools—and particularly Physical Education (PE)—provide a privileged setting to implement feasible and motivating strategies aimed at increasing physical activity and counteracting sedentary behaviors. Among these strategies, high-intensity interval training (HIIT) has emerged as a particularly time-efficient (low-volume) and curriculum-adaptable option, with robust evidence supporting its benefits in youth populations [3]. HIIT has demonstrated its potential across different populations to improve cardiorespiratory fitness (CRF) [4,5] and muscular strength [6,7], reduce body fat percentage [8,9], and improve BMI [10], among other benefits.

Existing studies provide key information on formats, dosing, and outcomes in school settings. In secondary education, Costigan et al. [11] demonstrated the feasibility of integrating HIIT blocks into the school day (8–10 × 30″:30″), resulting in improvements in CRF and reductions in BMI. Additionally, their program contributed to the consolidation of physical activity habits, with increases in daily moderate-to-vigorous physical activity measured by accelerometry [12].

At the anthropometric level, several controlled trials in overweight children have reported reductions in body fat percentage and waist circumference, as well as increases in CRF, when strength-related and plyometric components are incorporated into HIIT protocols [13,14,15,16,17]. Similarly, in primary education, high-intensity game-based approaches have shown effectiveness in improving health-related indicators, along with having good acceptability among students [18,19].

Thus, HIIT emerges as an effective strategy to improve physical fitness and anthropometric outcomes in children and adolescents with different health and weight profiles, with additional benefits in relation to blood pressure, lipid profile, and glycemic markers, particularly in youth with overweight/obesity or metabolic disorders [3,20].

Based on these studies, it is possible to derive design principles applicable to primary education: (1) very brief sessions (typically ≤10–12 min) with a work-to-rest ratio close to 1:1 and intensity control (≥85–90% HRmax); (2) the use of bodyweight/plyometric exercises and games to maximize practice density, reduce logistical barriers, and sustain motivation; (3) 2–3 sessions per week over 6–12 weeks; and (4) a flexible pedagogical organization that optimizes motivation and self-regulation of effort without the need for costly equipment [4,5,12,21,22,23,24,25,26].

However, HIIT is inherently intense and requires a high level of engagement and motivation from participants. This may pose a challenge in educational settings when attempting to sustain such protocols over the medium-to-long term, making it necessary to explore strategies that enhance adherence and motivation among students. In this context, some studies have explicitly implemented cooperative or paired formats in children and adolescents [4,12,23], while others have used individual execution with bodyweight exercises [5]. However, these formats (paired vs. individual) have not been directly compared under identical experimental conditions within the same age group. Given that the social component may influence motivation, self-regulation of effort, and adherence, it is relevant to examine this issue empirically in primary education.

Accordingly, the aim of the present study was to examine the impact of an individual and paired HIIT protocol based on functional bodyweight exercises on physical fitness-related and anthropometric outcomes in a population of primary school children. This protocol will be carried out during Physical Education lessons. Whereas in the individual protocol the exercises would be performed alone, in the partner protocol they would be performed in unison, thereby differentiating the motor and coordination demands. The following outcomes were hypothesized: H1: the individual HIIT protocol will show a significant improvement in VO_2_max (≥5% increase from baseline), whereas the paired HIIT protocol will show no significant change; H2: handgrip strength and standing long jump will improve significantly in the individual but not paired HIIT; and H3: the rating of perceived exertion will remain in individual but will decline in paired HIIT.

## 2. Materials and Methods

### 2.1. Design

A 10-week longitudinal experimental study with a pre-test–post-test design and three parallel groups was conducted (February–April 2025). Week 1 was devoted to familiarization with the high-intensity interval training (HIIT) methodology, week 2 to baseline assessments (pre-test), weeks 3–9 to the intervention, and week 10 to the final assessments (post-test). Experimental Group 1 (EG1) performed HIIT based on functional bodyweight exercises individually during the warm-up phase of Physical Education (PE) classes; Experimental Group 2 (EG2) completed the same HIIT protocol in pairs; and the Control Group (CG) performed standard PE warm-ups (without HIIT). This approach—very short micro-sessions integrated as a warm-up in PE and structured in a Tabata-like format (very brief intervals with high density), including the paired format—is consistent with recent trials and syntheses in school settings [3,5,27,28].

### 2.2. Participants

Sixty-one sixth-grade primary school children (11–12 years) from a public school, with no prior HIIT experience, participated in the study. Participants were allocated to the three study groups (EG1: *n* = 21, 11 girls, ten boys, 46.55 ± 10.23 kg, 11.54 ± 0.24 years, and 150.95 ± 7.02 cm; EG2: *n* = 20, 12 girls, eight boys, 11.53 ± 0.26 years, 40.37 ± 7.23 kg, and 149.3 ± 5.8 cm; CG: *n* = 20, 11 girls, nine boys, 11.62 ± 0.28 years, 45.48 ± 11.91 kg, and 153.00 ± 7.72 cm) using simple randomization with a 1:1:1 ratio. The random sequence was generated using a computer-based random number generator by a member of the research team who was not involved in baseline testing. Group allocation was performed after the completion of all pre-test assessments.

Due to the practical constraints of the school setting, formal allocation concealment was not implemented. However, baseline measures were obtained prior to group assignment to minimize potential allocation bias. No a priori sample size calculation was performed. The final sample size was determined by school availability and organizational constraints inherent to field-based interventions in primary education. Attendance of the intervention sessions was monitored throughout the study. All participants included in the analyses completed the intervention and post-test assessments.

All the study’s characteristics and objectives were explained to participants and their legal guardians. Participation was voluntary, and written informed consent was obtained from guardians prior to data collection and training. The anonymity of participants and their data, as well as the right to withdraw at any time, were ensured.

The inclusion criteria were enrollment in sixth grade, provision of informed consent, and commitment to complete the intervention. The exclusion criteria were students who were repeating sixth grade or presenting any clinical condition that contraindicated participation in HIIT.

The selection of this age group and school context was based on previous studies demonstrating good feasibility and responsiveness to intervention [25,26,28]. All procedures were conducted in accordance with the Declaration of Helsinki. The study was approved by the Ethics Committee of the Faculty of Education and Sport Sciences at the University of Vigo (reference number CP-310325-44; approval date: 11 April 2025).

### 2.3. Procedure

The 10-week fieldwork was conducted at the school facilities: the playground (physical fitness assessments), sports hall (HIIT sessions), and classrooms (anthropometric assessments). Pre- and post-test measurements were performed under the same instrumental and personnel conditions to minimize bias. The study timeline is presented below (Table 1).

**Figure 1:**
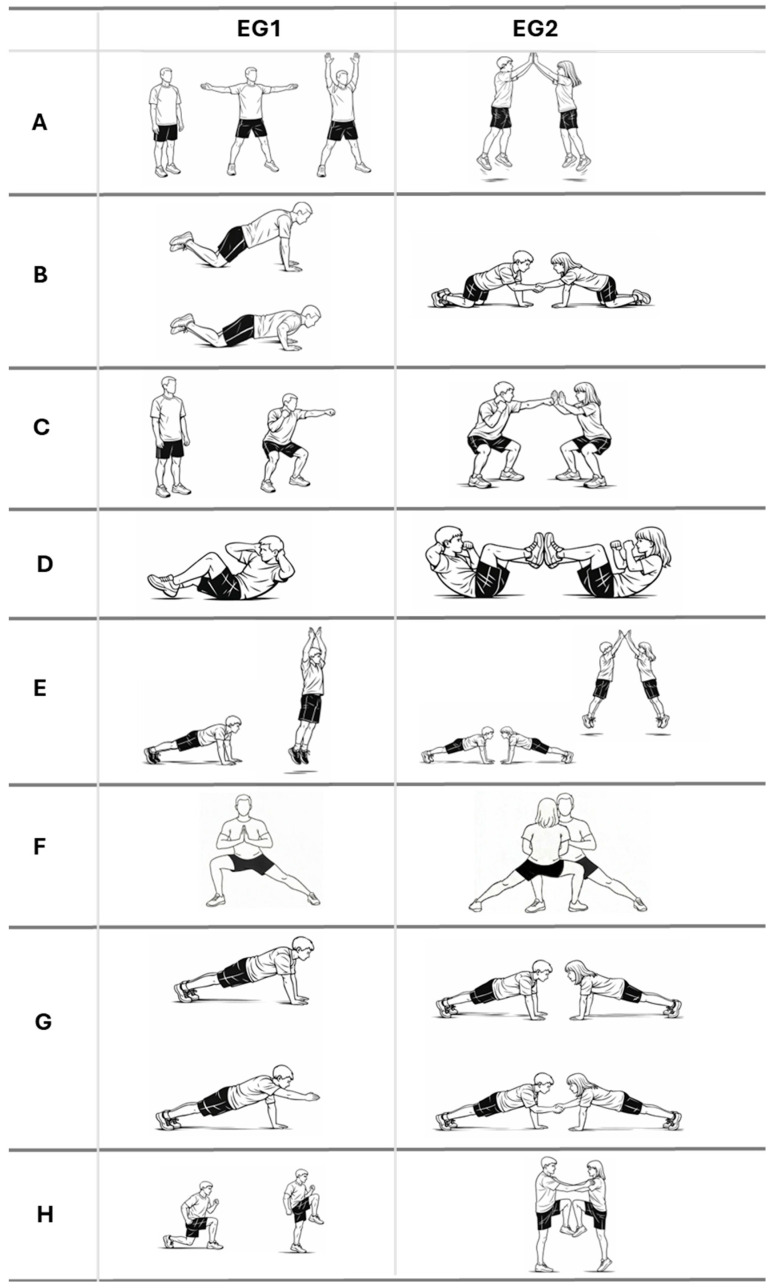
Exercises included in the individual (EG1) and paired (EG2) high-intensity interval training (HIIT) protocols. (**A**): *Jumping jacks*. Participants perform consecutive jumps while simultaneously opening and closing their arms and legs. In EG2, participants touch their partner’s hands overhead. (**B**): *Knee push-ups*. Participants perform push-ups with their knees supported on the ground. In EG2, partners touch hands at the end of each repetition. (**C**): *Squat and box*. Participants perform consecutive squats while striking with the upper limbs. In EG2, one participant strikes their partner’s palms; roles are switched in the second HIIT set. (**D**): *Abdominal crunches with rotation*. Participants perform abdominal crunches with trunk rotation while extending the opposite leg. In EG2, participants place the soles of their feet together and perform the movement synchronously. (**E**): *Burpee without elbow flexión*. Participants perform a burpee with a jump, without elbow flexion. In EG2, participants face each other and clap hands during the jump. (**F**): *Lateral lunge*. Participants perform alternating lateral lunges. In EG2, participants face each other, moving in opposite directions and touching hands. (**G**): *Front plank*. Participants perform a front plank while alternately raising one arm. In EG2, participants touch hands when raising the arm. (**H**): *Plyometric lunge*. Participants perform lunges with a knee drive. In EG2, both participants consecutively raise the same leg; they switch legs in the second set.

During week 1, familiarization and training were conducted, including a practical demonstration of the eight bodyweight exercises comprising the HIIT protocol (individual execution, EG1; paired execution, EG2; Figure 1). For EG2, pairs were formed by researchers and educators based on their perceived similarity in terms of physical fitness levels, without the use of formal matching criteria, and both participants were instructed to perform the exercises simultaneously during the entire 20 s work interval, without alternating turns or with planned rest within the interval. Low-intensity practice trials were also performed to consolidate technique and coordination.

The control group continued performing the standard warm-up activities routinely implemented in Physical Education lessons, representing an active comparison condition. Detailed session logs for these activities were not available, and their intensity was not objectively monitored.

Additionally, two tools were introduced to assess the process. One assessed the rating of perceived exertion (RPE), helping students determine whether they maintained optimal intensity during HIIT. Perceived exertion was assessed using a simplified 1–10 RPE scale, where 1 represented “very, very easy” and 10 represented “maximal effort”, (they were instructed to remain at ≥8). Previous research has shown that children aged 8–12 years are able to provide valid ratings of perceived exertion when using simple numerical or pictorial scales, with particularly strong validity reported from 10 to 11 years onwards [29]. Accordingly, RPE was used in the present study as a practical indicator of the perceived internal load during HIIT sessions in a school setting.

The second tool related to motivation, which was assessed using a pragmatic, non-validated single-item five-point Likert-type measure (1 = none; 5 = maximum). This single-item measure was included as a pragmatic indicator of students’ subjective experience during the HIIT sessions and was intended for process evaluation rather than as a comprehensive assessment of motivation. Similar brief measures have been used in school-based HIIT studies to monitor intervention reception and Fidelity [30].

Both instruments were administered during sessions 1, 7, and 14 of the HIIT protocol to monitor the process.

The intervention (weeks 3–9; 2 sessions/week; and 14 sessions) consisted of a 4 min Tabata protocol (8 × 20″ work/10″ rest) [31], performed in two sets per session (≤8 min of total high-intensity work including recovery intervals), with 1 min of recovery between sets. Prior to this protocol, a standardized warm-up was conducted, consisting of 5 min of continuous running and joint mobility exercises. In EG1, exercises were performed individually; in EG2, they were performed in pairs, while the CG performed the regular PE warm-up for that session. Music and verbal cues were used to enhance motivation and intensity.

Both experimental groups followed the same external interval structure (Tabata-type protocol: 8 × 20″/10″; two sets per session), but differed in task constraints. Exercises were selected to target large muscle groups and allow high work density, in line with previous school-based HIIT studies [5]. In the individual condition, exercises were performed independently, whereas in the paired condition, partner interaction and synchronization were required (Figure 1). Participants in the control group followed the standard Physical Education warm-up routinely implemented by the teachers. This typically consisted of 5–10 min of low-to-moderate intensity activities, including continuous running, joint mobility exercises, and preparatory games, without structured high-intensity intervals or specific intensity targets. All three groups carried out their protocol indoors.

### 2.4. Variables and Instruments

Two assessment sessions were conducted: pre-test (week 2) and post-test (week 10). The protocol and context were standardized and carried out under similar conditions by the same researchers to minimize bias.

Anthropometry: Body mass and height (Holtex stadiometer, ARRET-ET09103; Aix-en-provence, France), BMI, and body fat percentage were assessed using bioelectrical impedance (Tanita Corporation BC-601; Tokyo, Japan), ensuring participants’ privacy (individual measurements in the classroom). Measurements were taken with participants barefoot and conducted in the morning.

Physical fitness: Cardiorespiratory fitness (CRF/VO_2_max) was estimated using the 20 m shuttle run test (Léger) with standardized audio, recording stage and maximal speed [32]. Lower-body strength was assessed using the standing long jump (two attempts; best score), and upper-body strength using a hydraulic handgrip dynamometer (Jamar 5030J1; Sammons Preston Rolyan, Bolingbrook, IL, USA: two attempts per hand; best value), following the Eurofit battery guidelines [33]. The dynamometer handle was set at the second position, and measurements were performed with participants in a standing position. This combination of tests (20 m shuttle run, handgrip, and jump) is commonly used and sensitive to change in HIIT interventions in schoolchildren [25,26,27,28].

In addition, process evaluation was documented, as recommended to enhance interpretation and transferability in school settings [3]. Accordingly, in sessions 1, 7, and 14, participants in EG1 and EG2 completed a measure of rating of perceived exertion (RPE; 1–10 scale) and motivation scale (1–5).

### 2.5. Statistical Analysis

The effects of the intervention were analyzed using a mixed approach of within- and between-group comparisons. First, pre–post changes within each group (EG1, EG2, and CG) were assessed using paired *t*-tests, reporting absolute change (Δ), 95% confidence intervals (95% CI), the percentage change, and the within-group effect size (Cohen’s dz).

Subsequently, between-group differences in change (post–pre) were examined using one-way analysis of variance (ANOVA), reporting F, degrees of freedom, *p*, and partial eta squared (η^2^*_p_*). When appropriate, pairwise comparisons were performed using Welch’s *t*-test, reporting mean differences (ΔMeans) with 95% CI, *p*, and the standardized effect size (Hedges *g*).

As a sensitivity analysis, ANCOVA models were conducted for VO_2_max and body fat percentage, using post-test values as the dependent variables, group as the fixed factor, and baseline (pre-test) values and sex as covariates. Finally, process indicators (RPE and enjoyment/motivation) were compared between experimental groups using Welch’s *t*-tests at sessions 1, 7, and 14. These analyses were considered exploratory process evaluations. No formal correction for multiple comparisons was applied; therefore, the results were interpreted cautiously and in conjunction with effect sizes and overall trends.

## 3. Results

The baseline characteristics of the participants are presented in Table 2. Groups were comparable at baseline for age, sex distribution, height, body mass, body fat percentage, VO_2_max, and handgrip strength (all *p* > 0.05). Small but statistically significant differences were observed for BMI and standing long jump performance (*p* < 0.05). As a sensitivity analysis, ANCOVA models were conducted for VO_2_max and body fat percentage, using post-test values as the dependent variable, group as the fixed factor, and baseline value and sex as covariates. These analyses yielded results consistent with the primary change-score analyses, confirming the robustness of the findings after adjusting for baseline differences.

Table 3 presents the within-group (pre–post) changes for all variables across groups.

Table 4 presents the between-group comparisons (EG1–EG2–CG) for pre–post changes.

To further account for baseline differences and to provide a more comprehensive between-group comparison, additional ANCOVA models were conducted for all outcome variables. In these analyses, the post–pre change score was used as the dependent variable, group as the fixed factor, and baseline value and sex as covariates. Adjusted means, F values, *p*-values, and effect sizes are presented in Table 5.

### 3.1. Cardiorespiratory Fitness (VO_2_max)

**Figure 2:**
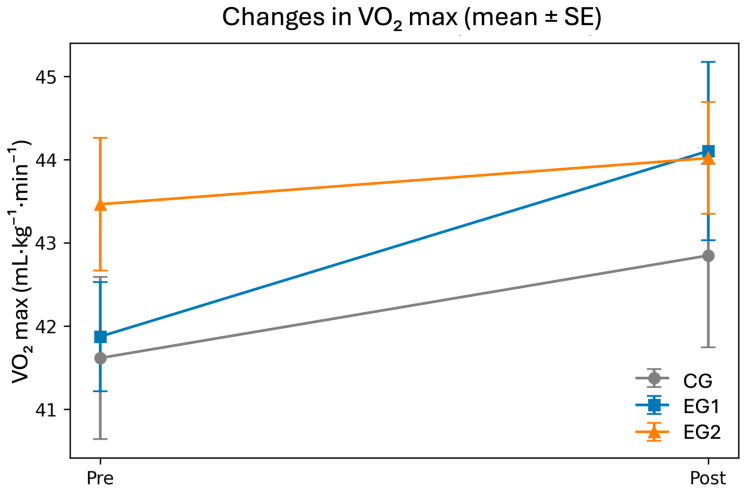
Changes in VO_2_max by group (error bars represent the mean ± standard error (SE).

In the within-group analysis, EG1 showed a significant increase in VO_2_max from pre- to post-test (Δ = +2.23 mL·kg^−1^·min^−1^; 95% CI (1.06, 3.40); *p* = 0.001; and dz = 0.87), CG exhibited a positive trend (Δ = +1.23; 95% CI (−0.04, 2.50); *p* = 0.056; and dz = 0.45), and EG2 showed a small, non-significant improvement (Δ = +0.55; 95% CI (−0.37, 1.48); *p* = 0.225; and dz = 0.28) (Figure 2). In relative terms, the mean changes were +5.32% (EG1), +2.96% (CG), and +1.28% (EG2).

The overall ANOVA on change scores for VO_2_max did not reach statistical significance (F(2, 58) = 2.44, *p* = 0.096, and η^2^_*p*_ = 0.078). However, exploratory pairwise comparisons suggested that the paired HIIT group improved less than the individual HIIT group (ΔMeans = −1.67; 95% CI (−3.12, −0.23); *p* = 0.025; Hedges g = −0.71; and 95% CI (−1.35, −0.08)). These findings should be interpreted with caution. Differences between EG1–CG (Δ = +1.00; *p* = 0.235) and EG2–CG (Δ = −0.68; *p* = 0.373) were not significant.

### 3.2. Body Fat Percentage and Body Mass Index

For body fat percentage, all groups showed significant reductions: CG (Δ = −2.00 *p.p*.; 95% CI (−3.60, −0.40); and *p* = 0.017), EG1 (Δ = −2.17 *p*.*p*.; 95% CI (−3.16, −1.18); and *p* = 0.001), and EG2 (Δ = −1.03 *p*.*p*.; 95% CI (−1.41, −0.64); and *p* = 0.001). In relative terms: −6.99% (CG), −7.96% (EG1), and −4.18% (EG2). The overall ANOVA was not significant (F(2, 58) = 1.36, *p* = 0.264, and η^2^_*p*_ = 0.045); however, the EG2–EG1 contrast showed a smaller reduction in EG2 (+1.15 *p*.*p*. vs. EG1; 95% CI (0.10, 2.19); *p* = 0.033; g = 0.68; and 95% CI (0.05, 1.31)). Comparisons between EG1–CG and EG2–CG were not significant (*p* ≥ 0.229). BMI showed no relevant between-group differences in change (ANOVA). However, a small but statistically significant increase was observed in EG2 from pre- to post-test (Δ = 0.17 kg/m^2^; 95% CI (0.002, 0.34); and *p* < 0.05), the magnitude of which is likely not clinically meaningful.

### 3.3. Strength

For handgrip strength, the ANOVA on change scores was significant (F(2, 58) = 4.05, *p* = 0.023; and η^2^_*p*_ = 0.123). Pairwise comparisons showed EG1 > CG (ΔMeans = +1.84 kg; 95% CI (0.11, 3.58); *p* = 0.038; g = 0.66; and 95% CI (0.03, 1.29)) and EG2 < EG1 (Δ = −1.84 kg; 95% CI (−3.28, −0.41); *p* = 0.013; g = −0.79; and 95% CI (−1.43, −0.16)). No differences were found between EG2 and CG (Δ ≈ 0.00 kg; *p* = 1.000). At the within-group level, only EG1 showed a significant increase in handgrip strength (Δ = +2.14 kg; 95% CI (0.85, 3.44); *p* = 0.003; and dz = 0.75).

For the standing long jump, the overall effect was not significant (ANOVA *p* = 0.297), although a significant within-group increase was observed in EG1 (Δ = +5.67 cm; 95% CI (0.50, 10.83); *p* = 0.033; and dz = 0.50). Because baseline differences were observed in standing long jump performance, this outcome was additionally analyzed using ANCOVA with the baseline value and sex included as covariates. After adjustment, no significant group effect was observed (Table 5), indicating that the descriptive pre–post differences were largely explained by baseline performance rather than by the intervention itself.

### 3.4. Process Indicators (Perceived Intensity (RPE) and Motivation)

**Figure 3:**
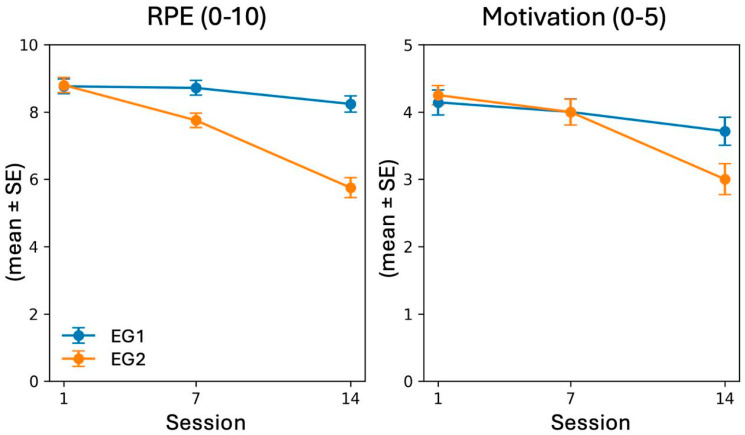
Trajectories of perceived intensity (RPE) and motivation (sessions 1, 7, and 14; mean ± standard error (SE)).

Perceived intensity (RPE) was similar in session 1 (EG1: 8.76 ± 1.00 vs. EG2: 8.80 ± 1.01; and *p* = 0.904), but was higher in EG1 in session 7 (8.71 ± 1.01 vs. 7.75 ± 0.97; and *p* = 0.003) and markedly higher in session 14 (8.24 ± 1.09 vs. 5.75 ± 1.33; and *p* = 0.000). For motivation, session 14 also favored EG1 (3.71 ± 0.96 vs. 3.00 ± 1.03; and *p* = 0.027) (Figure 3). These patterns are consistent with the observed differences in VO_2_max and strength: perceived exertion remained relatively stable across sessions in the individual HIIT group, whereas it declined in the paired group. As perceived exertion represents an indirect indicator of internal load, these findings may suggest differences in perceived training stimulus.

## 4. Discussion

The present study compared the effects of a functional HIIT protocol performed individually (EG1) versus in pairs (EG2) on physical fitness and body composition indicators in sixth-grade primary school children. The findings indicate that individual execution was associated with within-group improvements in cardiorespiratory fitness and strength, whereas the paired format was associated with smaller adaptations and a progressive decline in perceived exertion. This allows the findings to be interpreted in light of the existing literature in this population.

### 4.1. Cardiorespiratory Fitness

In our study, EG1 showed a significant improvement in estimated VO_2_max (+2.23 mL·kg^−1^·min^−1^; dz = 0.87), whereas EG2 exhibited a small, non-significant change (+0.55 mL·kg^−1^·min^−1^; dz = 0.28). The magnitude of change in EG1 (≈+5%) is consistent with several high-quality trials in schoolchildren. For example, Baquet et al. [25] reported improvements of 4–10% in VO_2_max after 10 weeks of 10–20 s intervals at 100–130% of maximal aerobic speed (MAS) in prepubertal children. Similarly, Nourry et al. [34] observed increases of up to +15.5% in VO_2_max after 8 weeks of intermittent HIIT in primary school children, with intensities at 100–130% MAS and short recovery periods, a stimulus pattern very similar to that used in EG1. McManus et al. [26] also demonstrated significant improvements in VO_2_max (+11.4 mL·kg^−1^·min^−1^) after 8 weeks of cycle ergometer sprint training in 10-year-old children, compared to a more modest increase in the continuous training group of the same study (+7.8 mL·kg^−1^·min^−1^).

Although the absolute increase in VO_2_max observed in this study was smaller than that reported in these interventions—likely due to duration of the HIIT protocol, the school-based context, or the use of bodyweight exercises—this pattern is consistent with our findings: when perceived intensity is maintained at the appropriated level, HIIT may be associated with cardiorespiratory improvements even with reduced volumes.

The EG1 > EG2 contrast may be interpreted in light of previous school-based HIIT studies [20], which suggest that maintaining sufficient perceived intensity may be relevant for CRF adaptations; however, the available evidence also indicates that the inadequate reporting of intervention fidelity is common, and reductions in intensity do not necessarily lead to null findings. Our process data are consistent with this pattern, with EG2 decreasing from an RPE ≈ 8.8 in session 1 to ~5.8 in session 14, whereas EG1 maintained values ≥8 throughout.

Moreover, several studies indicate that an intensity ≥90% HRmax or equivalent to MAS is required to elicit significant improvements in VO_2_max. This is supported by van Bjorn et al. [35], who found that intervals performed at >80% HRmax led to greater VO_2_max improvements than those observed in an active walking control group, while lower intensities failed to produce robust cardiorespiratory adaptations. However, as heart rate was not directly measured in the present study, these thresholds cannot be confirmed and should be interpreted as contextual reference values.

It should also be noted that the control group showed a near-significant improvement in VO_2_max (+2.96%, *p* = 0.056). This change may reflect familiarization effects associated with repeated testing, as well as normal growth- and maturation-related improvements in cardiorespiratory fitness at this age. Such effects have been reported previously in school-based studies and should be considered when interpreting between-group differences.

Therefore, our findings suggest that maintaining a high perceived intensity may be relevant for HIIT adaptations. The paired format involved additional interaction and coordination demands that may have influenced perceived exertion; however, the specific mechanisms underlying these differences (e.g., social pacing, waiting times, or changes in movement synchrony) were not directly measured and should be considered plausible hypotheses rather than demonstrated effects.

### 4.2. Strength

The strength results showed a pattern similar to that observed for CRF: EG1 showed significant within-group improvements in handgrip strength (+2.14 kg; dz = 0.75) and standing long jump (+5.67 cm; dz = 0.50), whereas EG2 showed minimal changes. These findings are consistent with other studies on functional and plyometric HIIT in schoolchildren.

In the study by Engel et al. [24], a functional micro-session HIIT program (squats, jumps, and locomotor tasks) significantly improved lower-body functional strength, demonstrating that short intervals performed at high execution speed have a marked neuromuscular effect. Similarly, Baquet et al. [25] reported increases in standing long jump after 10 weeks of short intervals at 100–130% MAS in primary school children, with moderate-to-large effect sizes in power-related outcomes. Evidence from studies in adolescents [17] also confirms that combining HIIT with plyometric training leads to significant improvements in lower-limb strength, with greater gains compared to HIIT without a plyometric component. Although our protocol involved bodyweight exercises and did not include advanced plyometrics, the high density of the Tabata protocol likely promoted sufficient neuromuscular recruitment to induce adaptations in EG1.

The lack of significant improvements in EG2 may be explained in the same way as for CRF: potential differences in perceived intensity and task execution characteristics may have contributed to the smaller adaptations observed in EG2, although repetition quality and volume were not objectively quantified. In the literature, trials with poor adherence to target intensity show weaker effects on strength, as reported in the meta-analysis by Liu et al. [20], particularly in interventions with declining RPE or uncontrolled rest periods.

However, although significant within-group improvements in strength were observed in the individual HIIT group, the overall between-group effects were limited. This was confirmed by the ANCOVA-adjusted analyses, which showed a significant group effect for handgrip strength but not for standing long jump after controlling for baseline differences (Table 5). These findings suggest that the strength adaptations observed should be interpreted cautiously and may reflect a combination of training exposure, maturation, and baseline differences.

### 4.3. Body Composition

All three groups reduced their body fat percentage, with a slightly greater decrease in EG1 (−2.17%) compared to EG2 (−1.03%) and CG (−2.00%). However, these differences were not significant between groups. This pattern is consistent with the available evidence in normal-weight children, where changes in body fat percentage following interventions ≤12 weeks are typically small.

Previous research indicates that HIIT is more effective in reducing adiposity in youth with overweight/obesity. For example, Racil et al. [13,15] reported greater reductions in body fat percentage and waist circumference when HIIT was applied in adolescents with obesity, particularly when combined with strength or plyometric training. Similarly, the meta-analysis by Li and Chen [36] found that reductions in BMI and body fat percentage after HIIT are mainly significant in obese populations, whereas results in healthy children are inconsistent. Liu et al. [37] reported a similar pattern, showing marked improvements in cardiometabolic parameters but modest changes in body composition in obese youth.

Therefore, the changes observed in our study—small and similar across groups—are consistent with this evidence. The lack of significant between-group differences may indicate that body composition is less sensitive to short-term, low-mechanical-load interventions in children without excess weight. The reduction in body fat percentage observed in the control group suggests that changes cannot be attributed solely to the HIIT intervention. Possible explanations include measurement variability associated with bioelectrical impedance analysis, particularly hydration status, the relatively active nature of CG warm-up, growth-related changes, although these factors cannot be disentangled in the present design.

### 4.4. Process Monitoring

A contribution of our study is the documentation of internal load (RPE) across multiple sessions. Most trials included in the review by Liu et al. [20] lack fidelity data, and those that report it tend to achieve better outcomes in CRF and strength. In our intervention, the decline in perceived exertion observed in the paired HIIT group co-occurred temporally with smaller adaptations in fitness outcomes.

Similarly, several previous studies highlight that the pedagogical organization of HIIT determines its effectiveness. In studies by Baquet et al. [25], van Bjorn et al. [35], Martínez-Vizcaíno et al. [19], and Engel et al. [24], researchers controlled interval structure, work-to-rest ratios, verbal instructions, and group dynamics, ensuring that all participants remained simultaneously active. When this level control is reduced, the reported benefits are often smaller.

Our results are consistent with this notion: the individual format was associated with higher perceived engagement across sessions, whereas the paired format involved additional interaction and coordination demands that may have been associated with a progressive reduction in perceived intensity. These observations do not provide direct evidence of differences in practice density or inactivity periods.

### 4.5. Individual vs. Paired High-Intensity Interval Training (HIIT) Implementation

Although some studies included in previous reviews have incorporated cooperative dynamics or game-based approaches—such as Ruiz-Ariza et al. [38], combining cooperative HIIT with cognitive improvements, or Martínez-Vizcaíno et al. [19], using high-intensity games—none have directly compared the same external structure delivered in individual versus paired formats within the same study and age group. Our design provides novel exploratory data in this regard.

Studies based on high-intensity games [18,19] report moderate improvements in CRF when group dynamics do not interfere with intensity, which contrasts with what was observed in our EG2. In these studies, the practice density is very high and all participants remain simultaneously active, avoiding “inactive time”. In contrast, in our protocol, the paired format involved additional coordinative and interaction demands. These demands may plausibly influence execution continuity and perceived exertion; however, micro-pauses, muscle activation, and movement quality were not directly measured and should therefore be considered hypothetical mechanisms rather than demonstrated effects. The lower adaptations observed in the paired group may be associated with social pacing and increased coordinative demands inherent to paired task execution; however, this interpretation remains speculative in the absence of objective measures of work density and internal load.

However, the authors acknowledge that the study had limitations that should be taken into account. First, allocation concealment was not formally implemented, which is common in school-based interventions but should be acknowledged as a limitation. In addition, pair allocation in the EG2 group was based on the practical judgment of researchers and educators to ensure similar physical fitness levels, without the use of formal matching criteria. This may have introduced some variability in baseline equivalence between paired participants. In this context, baseline differences were observed for standing long jump performance. Although this outcome was subsequently analyzed using ANCOVA with baseline value and sex included as covariates, residual confounding related to initial performance levels cannot be completely excluded. Therefore, results for standing long jump should be interpreted with caution and considered exploratory.

Second, the paired HIIT condition differed not only in social organization but also in motor and coordinative demands (e.g., synchronization and partner interaction), which may have influenced movement velocity and perceived internal load despite identical interval durations. Additionally, exercise intensity was not objectively monitored (e.g., via heart rate), which limits the ability to confirm whether the intended intensity was consistently achieved across participants. The assessment of motivation relied on a brief, non-validated single-item scale. Although this approach was chosen to maximize feasibility in a school setting, future studies should consider the use of validated multidimensional instruments (e.g., PACES) to more comprehensively assess motivational responses in children. Moreover, self-reported motivation may have been influenced by social desirability bias. Biological maturation status was not assessed. Given the age range of the participants (11–12 years), interindividual differences in maturation may have influenced strength, body composition, and cardiorespiratory adaptations. This limitation is inherent to many school-based field studies and should be considered when interpreting the results. Habitual physical activity outside school was not measured. Differences in extracurricular sports participation or daily physical activity may therefore have contributed to variability in the outcomes and cannot be ruled out. VO_2_max was estimated using a field-based test rather than directly measured, which may affect the precision of cardiorespiratory fitness assessment. Furthermore, the study was conducted in a single school, which may limit the generalizability of the findings. Finally, the relatively short intervention duration (10 weeks) may have constrained the magnitude of adaptations observed. Finally, study should be interpreted as exploratory, given the absence of an a priori sample size calculation and the relatively small number of participants per group and non-significant findings should be interpreted with caution.

## 5. Conclusions

This exploratory school-based study compared two pedagogical implementations of a brief functional HIIT protocol integrated into Physical Education lessons. The findings suggest that individual execution may have been associated with greater within-group improvements in cardiorespiratory fitness and strength, whereas the paired format was associated with smaller adaptations and a progressive decline in perceived exertion across sessions.

However, these findings should be interpreted with caution. The two experimental conditions differed not only in social organization but also in task constraints; furthermore, intensity was inferred primarily from perceived exertion, and no a priori sample size calculation was performed. In addition, the overall between-group effect for VO_2_max was not statistically significant. Moreover, because the two experimental conditions differed not only in social organization but also in motor and coordinative task demands, the present findings do not allow isolation of the effect of individual versus paired execution alone.

Taken together, the results suggest that pedagogical design and sustained engagement may be relevant for the implementation of brief HIIT activities in Physical Education. However, interpretations regarding underlying mechanisms remain speculative, as objective measures of internal load, activity patterns, or movement quality were not collected. Further research using larger samples and objective measures of internal load is therefore required before drawing firm conclusions about the relative effectiveness of individual versus paired formats.

From a practical perspective, the findings of this exploratory study offer several considerations for Physical Education (PE) teachers and practitioners working in school settings. Very short functional HIIT protocols (≤8 min) can be feasibly integrated into PE lessons as part of the warm-up, without disrupting curricular content, and may be associated with beneficial fitness responses when sufficient perceived intensity is maintained.

The results suggest that the individual execution of HIIT tasks was associated with higher perceived exertion across sessions than the paired format in this specific context. When paired or cooperative formats are used, teachers may need to carefully structure tasks to minimize inactive time or excessive coordination demands that could reduce movement density and perceived exertion.

Simple monitoring tools such as rating of perceived exertion (RPE) may be used as a pragmatic strategy to help students self-regulate effort during HIIT activities, while recognizing that RPE represents an indirect indicator of internal load.

Overall, these findings highlight the importance of pedagogical design in school-based HIIT. Prioritizing continuous engagement, clear task instructions, and basic intensity monitoring may support the implementation of HIIT activities within the time constraints of Physical Education lessons, although further research using objective measures is required before drawing firm conclusions about relative effectiveness.

## Figures and Tables

**Table 1:** Basic timeline of the experimental phase.

Week									
1	2	3	4	5	6	7	8	9	10
EG1 Familiarization	Pre-test	Individual HIIT-based warm-up. Twice per week.							Post-test
		S1: RPE-Motivation			S7: RPE-Motivation			S14: RPE-Motivation	
EG2 Familiarization		Paired HIIT-based warm-up. Twice per week.							
		S1: RPE-Motivation			S7: RPE-Motivation			S14: RPE-Motivation	
CG		Standard PE warm-up activities (active comparison condition). Twice per week							

RPE = assessed rating of perceived exertion; S = session.

**Table 2:** Baseline characteristics of participants.

Groups	GE1 (*n* = 21)	GE2 (*n* = 20)	GC (*n* = 20)
Sex (girl/boys)	11/10	12/8	11/9
**Variables**	(M ± SD)	(M ± SD)	(M ± SD)
Height (cm)	150.95 ± 7.02	149.30 ± 5.80	148.70 ± 8.28
Body mass (kg)	46.55 ± 10.23	40.37 ± 7.23	44.17 ± 8.11
BMI (kg·m^−2^)	20.29 ± 3.54	18.49 ± 2.72	21.07 ± 3.09
Body fat (%)	27.28 ± 5.47	24.50 ± 4.51	28.61 ± 6.36
VO_2_max (mL·kg^−1^·min^−1^)	41.87 ± 3.01	43.46 ± 3.57	41.62 ± 4.35
Handgrip strength (kg)	21.24 ± 4.53	20.50 ± 2.69	21.65 ± 4.90
Standing long jump (cm)	138.24 ± 14.97	139.65 ± 16.27	124.95 ± 24.16

BMI = body mass index; M = mean; and SD = standard deviation.

**Table 3:** Within-group (pre–post) changes for all variables.

Variable	Group	*n*	Pre (M ± SD)	Post (M ± SD)	Δ (95% CI)	Δ%	*p*	dz
VO_2_max	CG	20	41.62 ± 4.35	42.85 ± 4.93	1.232 (−0.04, 2.50)	2.96%	0.056	0.454
	EG1	21	41.87 ± 3.01	44.10 ± 4.90	2.229 (1.06, 3.40)	5.32%	0.001	0.866
	EG2	20	43.46 ± 3.57	44.02 ± 3.00	0.554 (−0.37, 1.48)	1.28%	0.225	0.280
Body fat (%)	CG	20	28.61 ± 6.36	26.61 ± 5.33	−2.000 (−3.60, −0.40)	−6.99%	0.017	−0.584
	EG1	21	27.28 ± 5.47	25.10 ± 4.65	−2.171 (−3.16, −1.18)	−7.96%	0.001	−0.999
	EG2	20	24.50 ± 4.51	23.48 ± 4.44	−1.025 (−1.41, −0.64)	−4.18%	0.001	−1.248
BMI	CG	20	21.07 ± 3.09	20.90 ± 2.98	−0.165 (−0.80, 0.47)	−0.78%	0.590	−0.123
	EG1	21	20.29 ± 3.54	20.20 ± 3.54	−0.081 (−0.80, 0.64)	−0.40%	0.816	−0.051
	EG2	20	18.49 ± 2.72	18.66 ± 2.78	0.170 (0.00, 0.34)	0.92%	0.047	0.474
Handgrip strength (kg)	CG	20	21.65 ± 4.90	21.95 ± 5.31	0.300 (−0.94, 1.54)	1.39%	0.617	0.114
	EG1	21	21.24 ± 4.53	23.38 ± 5.61	2.143 (0.85, 3.44)	10.09%	0.003	0.752
	EG2	20	20.50 ± 2.69	20.80 ± 3.21	0.300 (−0.38, 0.98)	1.46%	0.368	0.206
Standing long jump (cm)	CG	20	124.95 ± 24.16	127.90 ± 23.26	2.950 (−1.82, 7.72)	2.36%	0.211	0.289
	EG1	21	138.24 ± 14.97	143.90 ± 13.67	5.667 (0.50, 10.83)	4.10%	0.033	0.500
	EG2	20	139.65 ± 16.27	140.35 ± 21.18	0.700 (−3.30, 4.70)	0.50%	0.718	0.082

BMI = body mass index; M = mean; SD = standard deviation; Δ = absolute change; 95% CI = confidence interval; Δ% = percentage change; *p* = paired *t*-test; and dz = Cohen’s d for paired samples (within-group effect size).

**Table 4:** Between-group comparisons in change (post–pre) for all variables.

Variable	Contrast	Δ Means	95% CI	*t*(df)	df	*p*	g (95% CI)	g_ Low	g_ High	n1/n2
VO_2_max (mL·kg^−1^·min^−1^)	EG1–CG	0.997	(−0.674, 2.669)	1.207	38.60	0.235	0.370	−0.248	0.988	21/20
	EG2–CG	−0.677	(−2.201, 0.846)	−0.903	34.76	0.373	−0.280	−0.903	0.343	20/20
	EG2–EG1	−1.675	(−3.122, −0.227)	−2.343	37.37	0.025	−0.713	−1.346	−0.081	20/21
Body fat (%)	EG1–CG	−0.171	(−2.005, 1.662)	−0.190	31.92	0.850	−0.059	−0.671	0.554	21/20
	EG2–CG	0.975	(−0.661, 2.611)	1.239	21.18	0.229	0.384	−0.242	1.010	20/20
	EG2–EG1	1.146	(0.101, 2.192)	2.255	25.84	0.033	0.678	0.047	1.309	20/21
BMI	EG1–CG	0.084	(−0.841, 1.009)	0.184	38.56	0.855	0.056	−0.556	0.669	21/20
	EG2–CG	0.335	(−0.311, 0.981)	1.076	21.68	0.294	0.333	−0.291	0.958	20/20
	EG2–EG1	0.251	(−0.481, 0.983)	0.711	22.16	0.485	0.213	−0.401	0.827	20/21
Handgrip strength (kg)	EG1–CG	1.843	(0.109, 3.577)	2.150	38.97	0.038	0.657	0.028	1.287	21/20
	EG2–CG	0.000	(−1.376, 1.376)	0.000	29.58	1.000	0.000	−0.620	0.620	20/20
	EG2–EG1	−1.843	(−3.276, −0.409)	−2.625	30.06	0.013	−0.793	−1.430	−0.155	20/21
Standing long jump (cm)	EG1–CG	2.717	(−4.089, 9.522)	0.808	38.87	0.424	0.247	−0.368	0.862	21/20
	EG2–CG	−2.250	(−8.277, 3.777)	−0.757	36.88	0.454	−0.234	−0.857	0.388	20/20
	EG2–EG1	−4.967	(−11.302, 1.369)	−1.588	37.08	0.121	−0.483	−1.105	0.138	20/21

BMI = body mass index; ΔMeans = mean difference in change; 95% CI = confidence interval; *t* = Welch’s *t*-test; df = degrees of freedom; and g = Hedges g (95% CI).

**Table 5:** ANCOVA results for post–pre changes adjusted for baseline value and sex.

Outcome	Group	Adjusted Mean (EMM)	SE	F (Group)	*p*-Value	ηp^2^
**VO_2_max (mL·kg^−1^·min^−1^)**	EG1	2.30	0.46	4.21	0.019	0.13
	EG2	0.72	0.48			
	CG	2.22	0.44			
**Handgrip strength (kg)**	EG1	2.05	0.42	5.87	0.005	0.17
	EG2	0.21	0.39			
	CG	1.28	0.41			
**Standing long jump (cm)**	EG1	5.21	2.41	1.23	0.300	0.04
	EG2	0.88	2.36			
	CG	3.14	2.28			
**Body fat (%)**	EG1	−2.04	0.36	6.02	0.004	0.18
	EG2	−1.01	0.33			
	CG	−0.83	0.35			
**Body mass (kg)**	EG1	−1.52	0.29	7.84	0.001	0.22
	EG2	0.58	0.31			
	CG	−0.07	0.28			
**BMI (kg·m^−2^)**	EG1	−0.10	0.18	0.48	0.620	0.02
	EG2	0.15	0.17			
	CG	0.23	0.19			

Adjusted means (estimated marginal means, EMMs) were obtained from ANCOVA models using post–pre change scores as the dependent variable, group as the fixed factor, and baseline value and sex as covariates. ηp^2^ = partial eta squared (effect size). EG1 = individual HIIT; EG2 = paired HIIT; and CG = control group.

## Data Availability

The data are not publicly available due to ethical restrictions related to the involvement of minors, including parental consent procedures that did not include provisions for public data sharing, and institutional policies of the participating school. However, the data may be made available from the corresponding author upon reasonable request and subject to approval by the relevant ethics committee.
